# Supplementary material for: Dietary and Nutrition Interventions for Breast Cancer Survivors: An Umbrella Review
Source: Nutrients. 2025 Dec 21;18(1):30. doi: 10.3390/nu18010030 (PMC12787949; doi:10.3390/nu18010030)

Supplementary Material

Table S1. Full search strategy for the various databases

**PubMed (via Medline)**	("breast neoplasms"[MeSH] OR "breast cancer"[tiab] OR "breast tumour"[tiab] OR "breast tumor"[tiab] OR survivor*[tiab])<br>AND<br>("diet"[MeSH] OR "diet therapy"[MeSH] OR "nutrition therapy"[MeSH] OR diet*[tiab] OR nutrition*[tiab] OR "dietary intervention"[tiab] OR "nutrition counselling"[tiab] OR "nutritional counseling"[tiab])<br>AND<br>("systematic review"[pt] OR "meta-analysis"[pt] OR "systematic review"[tiab] OR "meta-analysis"[tiab])
**EMBASE**	('breast cancer'/exp OR 'breast cancer':ti,ab OR survivor*:ti,ab)<br>AND<br>('diet'/exp OR 'diet therapy'/exp OR 'nutrition'/exp OR diet*:ti,ab OR nutrition*:ti,ab OR 'dietary intervention':ti,ab)<br>AND<br>('systematic review'/exp OR 'meta analysis'/exp OR 'systematic review':ti,ab OR 'meta-analysis':ti,ab)
**CINAHL**	(MH "Breast Neoplasms" OR "breast cancer" OR survivor*)<br>AND<br>(MH "Diet Therapy" OR MH "Nutrition Counseling" OR diet* OR nutrition*)<br>AND<br>(MH "Systematic Review" OR MH "Meta Analysis" OR "systematic review" OR "meta-analysis")
**PsycINFO**	("breast cancer" OR survivor*)<br>AND<br>(diet* OR nutrition* OR "dietary intervention" OR "nutrition counselling" OR "nutrition counseling")

	AND<br>("systematic review" OR "meta-analysis")
**Scopus**	(TITLE-ABS-KEY("breast cancer" OR "breast neoplasm*" OR survivor*))<br>AND<br>(TITLE-ABS-KEY(diet* OR nutrition* OR "diet therapy"<br>OR "nutrition counselling" OR "nutritional counseling"))<br>AND<br>(TITLE-ABS-KEY("systematic review" OR "meta-analysis"))
**Cochrane Library**	("breast cancer" OR "breast neoplasm*" OR survivor*)<br>AND<br>(diet OR nutrition OR "diet therapy" OR "nutrition counselling"<br>OR "nutritional counseling")

Table S2. Methodological quality of the included reviews according to AMSTAR-2 tool

AMSTAR-2 Domain	Spark 2012	Amireault 2016	Burden 2019	Barchitta 2020	Shaikh 2020	Chen 2023	Buro 2024	Ryding 2024	Ng 2025
1. PICO components defined	✓	✓	✓	✓	✓	✓	✓	✓	✓
2. Protocol registered *C*	✗	✗	✓	✗	✓	✗	✗	✓	✓
3. Justification for study design	✗	✗	✓	✗	✓	✓	✓	✓	✓
4. Comprehensive search *C*	✓	✓	✓	?	✓	✓	✓	✓	✓
5. Study selection in duplicate	✗	✗	✓	✗	✓	✓	✓	✓	✓
6. Data extraction in duplicate	✗	✗	✓	✗	✓	✓	✓	✓	✓
7. List of excluded studies	✗	✗	✓	✗	✓	✗	✗	✓	✓
8. Adequate description of included studies	✓	✓	✓	✓	✓	✓	✓	✓	✓
9. Risk of bias (RoB) assessment *C*	✗	✗	✓	✗	✓	✓	?	✓	✓
10. RoB incorporated into interpretation *C*	✗	✗	✓	✗	✓	?	?	✓	✓
11. Methods for meta-analysis appropriate	✓	✓	✓	✓	✓	✓	✓	✓	✓
12. RoB assessed for meta-analysis	✗	✗	✓	✗	✓	?	✗	✓	✓
13. Heterogeneity explained	✗	✗	✓	?	✓	✓	✓	✓	✓
14. Investigated publication bias	✗	✗	✓	✗	✓	✗	?	✓	✓
15. Conflict of interest reported *C*	✓	✓	✓	✓	✓	✓	✓	✓	✓
**Overall Confidence**	**Low**	**Low–Moderate**	**High**	**Low**	**High**	**Moderate**	**Moderate**	**High**	**High**

Figure S1. Corrected Covered Area (CCA) Matrix

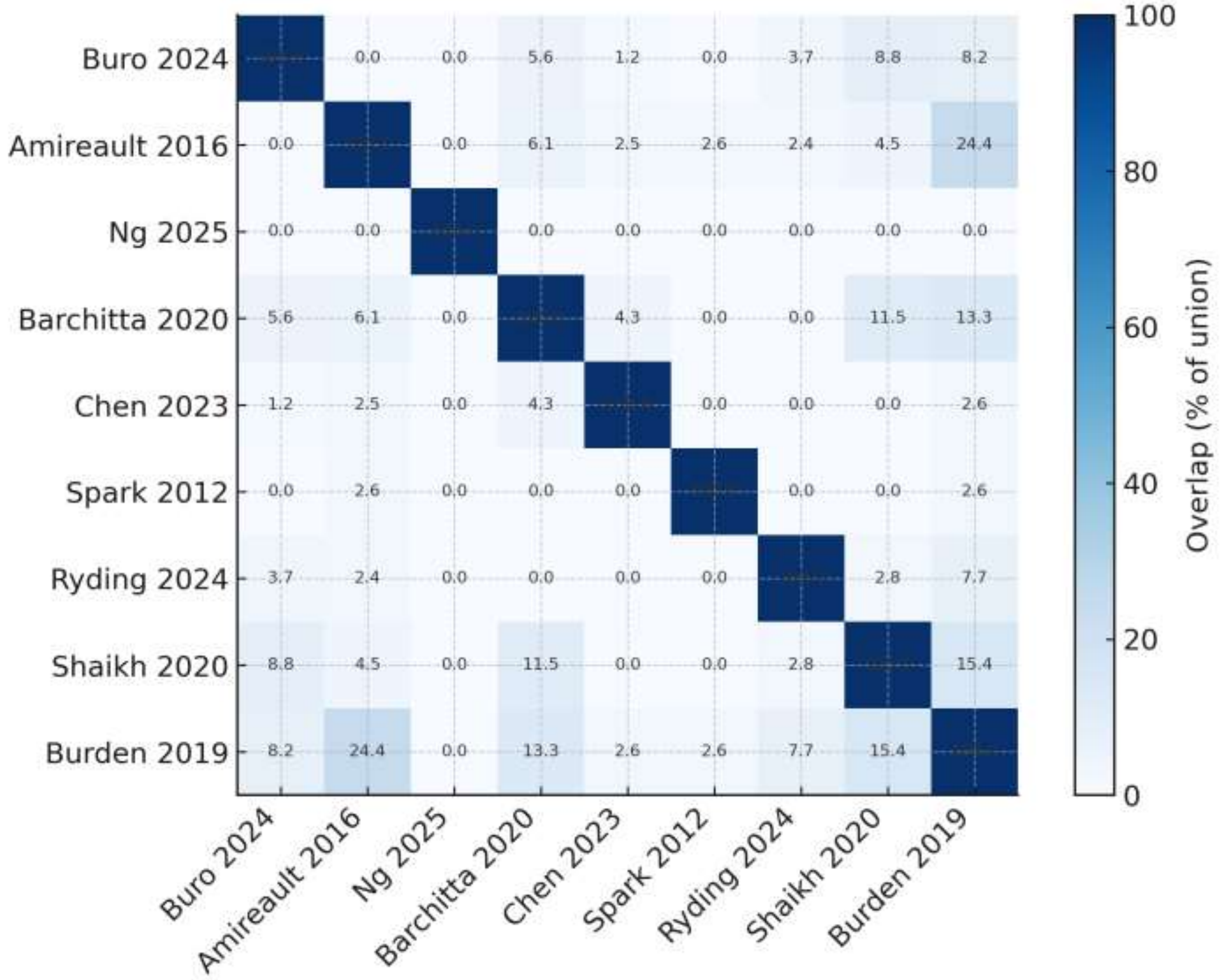

Supplement: Supplementary file 1 [file nutrients-18-00030-s001.zip › nutrients-4024754-supplementary.pdf]
